# Preservation of Colored Anatomical Specimens

**Published:** 1889-12

**Authors:** 


PRESERVATION OF COLORED ANATOMICAL SPECIMENS.—Alcohol changes and often destroys the coloring matters of these objects. Mr. Fabre-Domergue proposes a syrup in accordance with the following formula: Syrup of glucose, of 25° B. (specific gravity, 1.210), 1,000 gm.; white glycerine, 100 gm.; methylic alcohol, 200 gm.; camphor, q. s. The glucose is dissolved in hot water; after cooling, the other articles are added, with "a few pinches" of powdered camphor. The liquor should be neutralized with a little soda or potash lye; it should then be filtered and a little camphor should be dusted over the surface.—*Am. Jour. Phar.*

